# Allergy to Uncommon Pets: New Allergies but the Same Allergens

**DOI:** 10.3389/fimmu.2013.00492

**Published:** 2013-12-30

**Authors:** Araceli Díaz-Perales, David González-de-Olano, Marina Pérez-Gordo, Carlos Pastor-Vargas

**Affiliations:** ^1^Center for Plant Biotechnology and Genomics (UPM-INIA), Pozuelo de Alarcón, Madrid, Spain; ^2^Allergy Unit, Hospital Universitario Fuenlabrada, Madrid, Spain; ^3^Institute of Molecular Applied Medicine (IMMA), CEU San Pablo School of Medicine, Madrid, Spain; ^4^Department of Immunology, IIS-Fundación Jiménez Díaz, Madrid, Spain

**Keywords:** allergens, albumins, lipocalins, pet allergy, uncommon pet

## Abstract

The prevalence of exotic pet allergies has been increasing over the last decade. Years ago, the main allergy-causing domestic animals were dogs and cats, although nowadays there is an increasing number of allergic diseases related to insects, rodents, amphibians, fish, and birds, among others. The current socio-economic situation, in which more and more people have to live in small apartments, might be related to this tendency. The main allergic symptoms related to exotic pets are the same as those described for dog and cat allergy: respiratory symptoms. Animal allergens are therefore, important sensitizing agents and an important risk factor for asthma. There are three main protein families implicated in these allergies, which are the lipocalin superfamily, serum albumin family, and secretoglobin superfamily. Detailed knowledge of the characteristics of allergens is crucial to improvement treatment of uncommon-pet allergies.

Especially in urban areas, daily exposure to domestic animals, has been described as a potential risk factor for the development of respiratory symptoms and allergic disease, and is an increasingly common problem (1). The most frequent pet allergy is allergy to cats and dogs. However, in recent years it has become more and more popular to have other animals as pets, so that the risk of exposure to new and unknown potential allergens increased. The incidence of allergy to uncommon pets – that is, pets other than cats, dogs, birds, or fish – is unknown because descriptions in the literature include only isolated cases or small series. Nevertheless, the number of scientific publications has increased significantly over the last 10 years (2). Despite the lack of statistics providing the total number of households with exotic or non-traditional pets, such pets are certainly kept in a significant percentage of households. The ever-growing list of exotic pets includes various animals such as rodents (mice, rats, hamsters, guinea pigs, chinchillas, gerbils, jerboas, etc.), other mammals (ferrets, pigs, monkeys), spiders (tarantulas), reptiles (snakes), and exotic birds (3).

Most animal allergens are spread through airborne particles, and these particles have been detected in some animal-free environments (4). Despite the large number of animal allergens described, most of them belong to a small number of families, which is important for the study of their allergenicity and cross-reactivity. Most of the major mammalian allergens belong to one of three families: the lipocalin superfamily, the secretoglobin superfamily, or the serum albumin (SA) family. Within these families, the most widely studied allergens are lipocalin-like proteins and SAs.

This review summarizes the knowledge of the most common exotic animals used as pets, the allergic symptoms they might cause, and the new allergens responsible for those reactions.

## Epidemiology and Clinical Features

The number of households with pets is progressively increasing^1^. Six out of every 10 Spanish households – 8.5 million homes – keeps at least one pet, according to a study carried out in 2009 by the Propet pet professional fair^2^. There are around 20 million pets in Spain: 5.5 million dogs, 4 million cats, more than 7 million birds, and around 4.5 million fish. Uncommon animals also taken as pets, such as small mammals, amphibians, and reptiles, form a group of approximately two million. These data are proportionally similar to those described by the American Pet Products Association’s (APPA) 2011–2012 National Pet Owner Survey. This increase is even bigger within the group of exotic or uncommon animals; indeed, until 2008 – the year the financial crisis began – the number of animals imported to our country to later be sold increased by more than 100% relative to the previous decade, according to the convention on international trade in endangered species of wild fauna and flora^3^. This considerable increase in ownership of uncommon pets might be due to trends in consumption or to a larger proportion of people living in small apartments, where having large animals is usually not permitted. As happens with traditional pets such as dogs and cats, sustained contact with these exotic animals can sometimes lead to the development of allergic symptoms.

The most frequently reported clinical features of allergy to uncommon animals are usually the result of inhalation, contact, or bites.

### Mammals

Exotic mammals are the largest group of uncommon pets. The most frequent symptoms presented after exposure to these animals – i.e., rhinitis, conjunctivitis, and asthma – affect the upper and lower respiratory tract and have been reported in relation to prairie dog (5), chinchilla (6), guinea pig (7, 8), ferret (9, 10), gerbil (11, 12), hamster (13–17), hedgehog (18), rabbit (19–21), hare (22), and monkey (23–25). Contact urticaria has also been reported with chinchilla (6), ferret (10, 26), and hedgehog (18). In cases the owner was bitten by the animal, the subsequent symptoms reported varied from urticaria (15, 27) or respiratory discomfort (28) to anaphylactic shock, as described with gerbil (28), hamster (14, 29, 30), sunda slow loris (2), and mouse (31).

### Reptiles

The main symptoms developing after exposure to these animals also affect the upper and lower respiratory tract; indeed, asthma, rhinitis, and conjunctivitis after exposure to iguana (25, 32–34) and lizard (2) have been documented. The symptoms reported after reptile bites range from eruption of crusty pruriginous papules after an iguana bite (35) to anaphylaxis with clear predominance of vascular symptoms after bites by lizard (36–38), crotalus (39), and king cobra (40), but the mechanism underlying these reactions was not studied.

### Birds

To a greater or lesser extent, mostly all the symptoms prompted by exposure to birds affect the respiratory tract. Besides rhinitis and/or asthma (41, 42), the inhalation of allergens related to exotic birds, and as it happens in cases of exposure to birds not considered as exotic, might sometimes cause hypersensitivity pneumonitis as described after exposure to lovebirds (43, 44), cockatiel (45), pheasants (46), canaries (47), parakeets (48, 49), parrots (50), geese (51), and owls (52). Other respiratory diseases, such as bronchiolitis obliterans due to parakeets, have also been described (53). Patients with bird-egg syndrome may present respiratory symptoms induced by bird antigens and gastrointestinal symptoms after the intake of bird egg (44, 54, 55).

### Others

Arachnids have recently come to be regarded as pets as well. Some arachnids, including tarantulas, have hairs that produce urticaria that is not immune-mediated and can result in persistent papular dermatitis, or, when the hairs come into contact with the cornea and conjunctiva, ophthalmia nodosa (56). Generalized rash and hypotension after a spider bite has also been reported (57), but no allergic study was carried out.

## Allergen Sources

Contrary to popular belief, dander, and not hair, is the main cause of allergy to animals. Dander contains allergens formed in the sebaceous gland secretions and saliva. In animals, as in humans, the skin sheds gradually as microscopic scales. Secretions containing allergens are adhered to hair and stratum corneum of the skin. Small particles are able to remain floating in the air for long periods of time and, when inhaled, cause allergic symptoms in the nose, eyes, and respiratory tract. These particles settle slowly on the floor or furniture and are re-dispersed in the air so that allergens can be inhaled although the pet is not present at the time. For this reason, patients notice animal-allergy symptoms just entering homes or places where there are, although not present at the time. While less frequent, hair can also cause allergy, though animal hair stays at the floor and is not present in ambient air. Few allergens have been described in uncommon pets, and most are homologous to the lipocalin family, the secretoglobins family, the family of immunoglobulins, and SAs.

## Allergens to Uncommon Mammalian Pets

Although there is a growing number of reports on allergens mediated by uncommon pets, there is little information about the allergens involved. We will now describe only the allergens already characterized within this family of pets. Not all of them are fully characterized and some of the allergens presented in Table 1 are only partially described and tentatively named. Rodents and other small domestic and laboratory animals have a high sensitizing potential (58). Allergenic proteins of small rodents have been found in hair, urine, and salivary gland extracts. In certain rodents, 10 or more allergens have been identified. The molecular weight of these allergens ranges from 8 kDa to more than 80 kDa. The major allergens of mouse (Mus m 1, Mus m 2), rat (Rat n 1A, Rat n 1B), and guinea pig (Cav p 1, Cav p 2) have all been identified, and extracts are commercially available for each animal. Rabbit allergens are not well described, but at least three individual glycoproteins, Ory c 1, Ory c 2, and Ory c 3 are identified in hair, dander, and urine (21, 59). Most of them are included in the lipocalin family (Table 1). For the Siberian hamster, no allergen has been officially named, although one has been recently sequenced (deposited in GenBank, accession number GI: KF148615). By contrast, IgE immunoblotting revealed three IgE-binding bands of about 18, 21, and 23 kDa which correspond to isoforms of a single allergen which has been identified as a lipocalin (29).

**Table 1:** **Uncommon pets allergens**.

Common name	Species	Source	Allergen	Family	Reference
Chinchilla	*Chincilla lanigera*	Epithelial, saliva, urine	Chi La	Protein kinase inhibitor	(6)
			Chi Lb	Lipocalin	
Guinea pig	*Cavia porcellus*	Epithelial, saliva, urine	Cav p 1^a^	Lipocalin	
			Cav p 2^a^	Lipocalin	
			Cav p 3^a^	Lipocalin	
			Cav p 4^a^	Serum albumin	
			Cav p 6^a^	Lipocalin	
Gerbil	*Meriones unguiculatus*	Epithelial, saliva, urine, sleep bed	Mer un 23kDa	Lipocalin	(12)
			Mer un 4	Serum albumin	(60)
Hamster	*Phodopus sungorus*	Epithelial, saliva, urine	Phos 21 kDa	Lipocalin	(29)
Rat	*Rattus norvegicus*	Epithelial, saliva, urine	Rat n 1^a^	Lipocalin	
			Rat n 4	Serum albumin	(61)
			Rat n 7	Immunoglobulin	(62)
Mouse	*Mus musculus*	Epithelial, saliva, urine	Mus m 1^a^	Lipocalin	
			Mus m 2	*Unknown*	(61)
			Mus m 4	Serum albumin	(61)
			Mus m 7	Immunoglobulin	(62)
Rabbit	*Oryctolagus cuniculus*	Epithelial, saliva, urine	Ory c 1^a^	Lipocalin	
			Ory c 2	Lipocalin	(59)
			Ory c 3^a^	Secretoglobin	
			Ory c 4^a^	Lipocalin	
Ferret	*Mustela putorius*	Epithelial, saliva, urine	Mus p 17	*Unknown*	(9)
			Mus p 66	Serum albumin	(9)
Pig	*Sus scrofa*	Meat	Sus s 5	Lipocalin	(63)
			Sus s 6	Serum albumin	(64)

*The allergen names delivered by IUIS Allergen Nomenclature Sub-Committee have been marked by ^a^*.

Other popular uncommon pets are small pigs, mini pigs, or teapot pigs. Pig allergy was studied from the point of view of food sensitization and occupational allergy. Pig hair and dander are important inducers of occupational allergies in cattle-exposed farmers (65, 66). More than 10 allergens have been associated with allergic events in patients who are caretakers of this animal. The most prevalent allergens include lipocalin proteins and albumins (Table 1)^4^.

The domestic ferret (*Mustela putorius furo*) is the third most common furred pet in US households. Some case reports have appeared in the literature. A 66-kDa and a novel 17 kDa protein were characterized as putative allergens in ferret extract prepared from fur, urine, feces, and bedding material (9, 10). The 66-kDa protein was assumed to represent ferret albumin because of its *in vitro* cross-reactivity with cat albumin. The novel 17 kDa showed molecular weight that was similar to lipocalin.

## Other Allergens in Uncommon Pets

Spiders have become pets because of their small size and ease of care. Most of the reported events regarding these animals are produced by toxicity. Little has been published on environmental spider allergy or allergy to spider bite. However, Bobolea and colleagues identified two new allergens in *Holocnemus pluchei*: hemocyanin and arginine kinase (67).

In reptiles, the primary IgE-binding proteins are present in the venom, urine, and epithelial cells. The principal allergens have been described among 59–63 and 8–15 kDa but have not been identified yet (68).

Although many allergens have been described in birds, all of them have been associated with ingestion and are not related to the role of birds as pets. Only in some reports which refer to allergy by inhalation, the molecular weights of allergens have been described but without their identification (44).

## Major Allergens in Uncommon Pets

Most of the allergens reported in cases of allergy to uncommon pets have been characterized by homology to other allergens previously described in meat or milk animals. Here we present a descriptive summary of the most relevant protein families identified as allergens such as the lipocalin family, SAs, secretoglobins, and other allergens, as described in Table 1. Within these families, the most widely studied allergens are lipocalin-like proteins and SAs.

### Lipocalins

Most of the important animal-derived allergens belong to the lipocalin protein family (Table 1). Lipocalin allergens are found in dander, saliva, and urine. These allergens disperse effectively and are widely present in indoor environments. Initially, lipocalins were characterized as transport proteins for principally hydrophobic molecules such as retinol, odorants, steroids, and pheromones, but now they are known to be involved in many other biological functions (69).

Lipocalins are a large group comprising proteins from vertebrate and invertebrate animals, plants, and bacteria. The family is part of a larger^5^ superfamily, calycins (70).

The amino acid sequences of lipocalins compromise 160–230 residues with an average predicted molecular mass of about 20,000 Da (without post-translational modifications) (70). They can be *N*- and/or *O*-glycosylated. The overall amino acid identity between lipocalins is 20–30%, but it can be considerably higher. For example, human lipocalin-9 is more than 50% identical to its rodent homologs, and identities of about 40% are found with Mus m 1, Rat n 1, Equ c 1, and Fel d 4. The amino acid identity of dog Can f 1 with human tear lipocalin is about 60%. Although the sequential identity among lipocalins is low in general, they share a common three-dimensional structure (70). The central β-barrel of lipocalins, which is composed of eight anti-parallel β-strands, encloses an internal ligand-binding site. Most lipocalins contain one or more intramolecular disulfide bonds.

The arrangement of lipocalin molecules in a multisubunit complex (oligomerization) is variable (71). In all, the physicochemical and structural features of the characterized lipocalin allergens are not known to account for their allergenic capacity or to distinguish them from other lipocalin proteins (72). However, they induce IgE production in a large proportion of atopic individuals exposed to the allergen source.

As lipocalins are known to carry small hydrophobic ligands in their internal ligand-binding site, recent studies finding that pollen extracts from birch and several other plants contain E1-phytoprostanes and possibly other Th2-deviating lipid mediators are of interest (73, 74). It has even been suggested that lipid binding can be a key characteristic for many allergens because lipids can directly activate innate immunity (73). Although there are no data supporting the idea that lipocalin allergens would carry immunomodulatory substances favoring allergy, the hypothesis is no doubt worth further examination.

There are only a few T cell epitopes reported for lipocalin allergens, and those examined have proved to be suboptimal. Moreover, the frequency of lipocalin allergen-specific CD4+ T cells is very low in the peripheral blood. Importantly, recent research suggests that the lipocalin allergen-specific T cell repertoires differ considerably between allergic and healthy subjects. These observations are compatible with the hypothesis that the way CD4+ T-helper cells recognize the epitopes of lipocalin allergens may be implicated in the severity of the symptoms (75).

### Serum albumin

Serum albumins, characterized by a molecular weight of 67 kDa and a tendency to participate in IgE-mediated cross-reactions, are recognized by the serum of 20–30% of patients with some pet allergy (Table 1) (76).

Serum albumin, often referred to simply as albumin, is a globular protein that in humans is encoded by the ALB gene (77). SA is the most abundant plasma protein in mammals. Albumin is essential for maintaining the oncotic pressure needed for proper distribution of body fluids between intravascular compartments and body tissues. It also acts as a plasma carrier by non-specifically binding several hydrophobic steroid hormones and as a transport protein for heme groups and fatty acids. Too much SA in the body can be harmful.

Allergic sensitization to SA can occur by inhalation as well as ingestion. SAs are found in dander and saliva of pets and are important inhalant allergens.

The best characterized member of this family is the bovine serum albumin (BSA). Its covalent bonds maintain its tertiary structure under denaturing conditions (e.g., low pH or heating). The protein is organized in three homologous domains (I–III) and consists of nine loops (three loops/each) connected by 17 covalent disulfide bridges. Most of the disulfide bonds are well protected in the core of the protein and are not readily accessible to the solvent (78). Interestingly, members of this family are important food allergens in bird and mammal species. However, there are no reports of sensitization to SA by inhalation in birds.

### Secretoglobin allergens

Secretoglobins are the most potent allergens in cat and there have been described as allergens in other pets (21). These proteins show unknown function, and they are produced by the skin and by salivary and lacrimal glands of pets (79). Secretoglobins are transferred to the pelt by licking and grooming. Dried saliva and dandruff are spread from the hair to the surrounding environment as small airborne particles possibly causing sensitization in susceptible individuals (79).

This family consists of two allergic relevant members to pets such as Fel d 1 and Ory c 3. Little is known about rabbit allergen, although on Fel d 1 there is more information. Fel d 1 is a protein produced largely in cat saliva and sebaceous glands. The complete quaternary structure of Fel d 1 has been determined. The allergen is a tetrameric glycoprotein consisting of two disulfide-linked heterodimers. Both chains share an all alpha-helical structure (80).

### Other allergens

Other allergens have been described in domestic animals (Table 1), and are included in the family of the caseins, immunoglobulins, gelatins, and transferrins. These are all minor allergens that are present in secretions (e.g., saliva, urine, and semen) and flaking of the animals.

## Concluding Remarks

Exotic pet allergy and their associated respiratory symptoms have increased in recent years. Nowadays, avoidance therapy is the best measure for the prevention of any pet allergic reaction. Biomolecular characterization of allergens remains essential to the development of emerging therapeutic modalities to treat respiratory symptoms, such as attenuated allergy vaccines.

This review compiles the existing descriptions of the main exotic or uncommon pets that cause allergy in our environment and the main allergens implicated. Most of the animal allergens described belong to a small number of families. Furthermore, it would be reasonable to study the allergenicity and cross-reactivity of these major pet allergens to improve specific treatment of patients with allergy to animals.

## Author Contributions

All author have contributed to the conception, design, and drafting of the paper.

## Conflict of Interest Statement

The authors declare that the research was conducted in the absence of any commercial or financial relationships that could be construed as a potential conflict of interest.

## Footnotes

^1^http://www.americanpetproducts.org/press_industrytrends.asp

^2^http://www.ifema.es/ferias/propet/default.html

^3^http://www.cites.org

^4^www.allergome.com

^5^http://prosite.expasy.org/PS00213
